# Optimisation of Wavelength Modulated Raman Spectroscopy: Towards High Throughput Cell Screening

**DOI:** 10.1371/journal.pone.0067211

**Published:** 2013-06-25

**Authors:** Bavishna B. Praveen, Michael Mazilu, Robert F. Marchington, C. Simon Herrington, Andrew Riches, Kishan Dholakia

**Affiliations:** 1 SUPA, School of Physics and Astronomy, North Haugh, University of St Andrews, St Andrews, Fife, Scotland, United Kingdom; 2 School of Medicine, Medical and Biological Sciences Building, University of St Andrews, North Haugh, St Andrews, United Kingdom; 3 Jacqui Wood Cancer Centre, University of Dundee, Ninewells Hospital, Dundee, United Kingdom; University of Navarra, Spain

## Abstract

In the field of biomedicine, Raman spectroscopy is a powerful technique to discriminate between normal and cancerous cells. However the strong background signal from the sample and the instrumentation affects the efficiency of this discrimination technique. Wavelength Modulated Raman spectroscopy (WMRS) may suppress the background from the Raman spectra. In this study we demonstrate a systematic approach for optimizing the various parameters of WMRS to achieve a reduction in the acquisition time for potential applications such as higher throughput cell screening. The Signal to Noise Ratio (SNR) of the Raman bands depends on the modulation amplitude, time constant and total acquisition time. It was observed that the sampling rate does not influence the signal to noise ratio of the Raman bands if three or more wavelengths are sampled. With these optimised WMRS parameters, we increased the throughput in the binary classification of normal human urothelial cells and bladder cancer cells by reducing the total acquisition time to 6 s which is significantly lower in comparison to previous acquisition times required for the discrimination between similar cell types.

## Introduction

Raman spectroscopy is a vibrational spectroscopic technique that provides information regarding the chemical composition of a sample of interest [1]. The technique is sensitive enough to detect changes in the chemical composition of the sample, which makes it a powerful diagnostic tool [2]. However Raman scattering is a relatively weak inelastic process with only 1 in 10^6^ photons contributing to the Raman signal. In addition, the weak Raman signal is obscured by the luminescence background resulting from the auto-fluorescence of the biological sample and the sample substrate [3]. Suppressing this fluorescence background would enhance the contrast between the diseased cells and normal cells, allowing better classification, which is desirable for clinical applications [3]–[5].

Numerous techniques have been demonstrated to reduce or suppress fluorescence background [6]–[11]. Among these, WMRS is straight forward to implement in terms of the instrumentation required and reliability, as this technique is independent of the polarisation and temporal properties of the signal. Although the general principle of this technique was demonstrated three decades ago [12]–[15], it was only recently that this method has evolved and been successfully adapted for biomedical applications [3].

WMRS exploits the fact that the Raman peaks shift along with a shift in the excitation wavelength (<1 nm), while the background remains a constant. Lock-in detection schemes or multivariate methods such as Principal Component Analysis (PCA) and Expectation Maximization (EM) algorithms can be used to extract the modulating Raman peaks, suppressing the broad fluorescence background [6], [16]. The outcome is a differential spectrum where only peaks corresponding to the Raman bands are visible.

Although the applicability of WMRS in the field of bio-medicine has been demonstrated, the modulation parameters remain to be optimized, which is the subject of this study. This optimisation is important since addition of fluorescence suppression techniques to standard Raman is generally considered as a time consuming process. We here demonstrate a systematic approach to typically optimise the WMRS parameters. Optimizing various parameters of WMRS is crucial to ensure enhancement in SNR of Raman bands which allows reduction of the acquisition time whilst retaining appropriate discrimination between biomedical samples. This is central for developing high throughput label-free cell screening methods such as Raman activated flow cytometry to classify normal and abnormal cells derived from body fluids such as blood and urine [17].

Here we systematically optimise various parameters of WMRS for biological samples (cells). We have optimized parameters such as the range within which the wavelength was modulated, time constant, number of cycles and sampling rate. The effectiveness of the optimization was confirmed by performing binary classification of normal human urothelial cells (SV-HUC-1) and bladder cancer cells (MGH-U1) with a total acquisition time as low as 6 s. While considering previous works on similar cell types, taking into account the scaling of Raman signal intensity inversely with λ^4^ (where λ is the excitation wavelength) and the time period to collect the Raman signal per cell, it can be noted that by optimising WMRS it is possible to reduce the total acquisition time significantly. Whilst we appreciate a direct one to one comparison is difficulty, our acquisition time, at a longer wavelengths than previously reported compares favourably with the 30 s required for cell discrimination shown in earlier work [18].

## Experimental Methods

### Instrumentation

The experiments were performed on a custom designed Raman microscope, similar to that reported elsewhere [19]. The laser source used was a tunable diode laser (Sacher, Littmann configuration, centred at λ = 785 nm, maximum power 1 W, total tuning range 200 GHz). The laser beam was expanded using a telescope to appropriately fill the back aperture of a microscope objective (Olympus, magnification 40x/NA = 0.74) after passing through a line filter. The back scattered Raman photons were collected through the same objective and coupled through a F/# matcher into the spectrometer which was equipped with a 400 lines/mm grating with a deep depletion, back illuminated and thermo-electrically cooled CCD camera (Newton, Andor Technology). The sample was illuminated with a standard Kohler illumination set-up in the transmission mode. A waveform/function generator (Keithley, 50 MHz) was connected to the tunable laser to modulate the wavelength. The average excitation power at the sample was maintained at ∼ 200 mW throughout the experiment.

### Cell Culture

The Normal Human urothelial cells (SV-HUC-1) had been immortalised by transformation with simian virus 40 (SV40) [20]. The SV-HUC-1 cells were cultured in the following medium: F-12 nutrient mixture with L-glutamine (Ham - GIBCO 21765), with added human insulin 5 µg/ml, hydrocortisone 1 µg/ml, transferrin 5 µg/ml, glucose 2.7 mg/ml (Sigma), non-essential amino acids 0.1 mM (Gibco), penicillin 100 µg/ml, streptomycin 100 units/ml (Sigma) and fetal calf serum 1% (Globepharm).

A cell line derived from a recurrent human bladder tumour (MGH-U1) was maintained in long-term culture [21]. MGH-U1 cells were cultured in the following medium: GIBCO D - MEM:F12 (1∶1) with added fetal calf serum 7% (Globepharm), penicillin 100 µg/ml, L-glutamine 2 mM and streptomycin 100 units/ml (Sigma).

### Sample Preparation

The sample chamber used for this experiment was built using a 80 µm deep vinyl spacer between a quartz slide of 1 mm thickness and a quartz cover slip of 150 µm thickness (SPI supplies, UK). 20 µl of the sample consisting of either Polystyrene beads or cells (MGH-U1/SV-HUC-1) was loaded into these chambers for analysis.

### Data Treatment

Principal Component Analysis (PCA) was used to obtain the differential Raman spectra from each set of acquired wavelength modulated Raman signals. The first principle component evaluated corresponds to the maximal variation between the spectra. This variation is induced by the discrete shift of the Raman peaks and gives the derivative-like differential Raman spectrum where the fluorescence is eliminated [16].

## Results and Discussion

In WMRS the key factors that may be optimised to improve the acquisition time are the modulation amplitude, sampling rate, time constant and number of cycles. The modulation amplitude refers to the range within which the wavelength was modulated while acquiring the Raman signal. In this present study, the wavelength was modulated discretely in a symmetric trapezoidal pattern, to ensure maximum distance between the two adjacent wavelengths chosen for modulation. Since each wavelength is sampled twice during a trapezoidal scan, it corresponds to two cycles. Sampling rate corresponds to the number of acquisition steps per cycle. The time constant is the single exposure time for acquiring a Raman spectrum. The total acquisition time is the product of the time constant, sampling rate and total number of cycles.

The natural frequency with which the probe molecule vibrates is affected by the intermolecular spacing, composition and kinetic energy of the adjacent molecules with which it interacts. The balance between the life-time of the coherent vibration of the molecules in the ground state and the time taken by the excited molecules to reach the ground state thus changes drastically between solids, liquids and gases leaving the line shape different in each of these cases. While the line shape in solids takes a *Gaussian profile* due to the statistical distribution of the environment, in gases it is *Lorentzian* and in liquids it is a combination of the Gaussian and Lorentzian profile (*G-L profile/voigt profile*) [22], [23]. Biological cells, which arguably exhibit both solid and liquid behavior, thus should differ in the optimal modulation parameter in comparison to standard Raman samples [24]. Due to the vast contribution from the numerous molecules in cells, the Full Width Half Maximum (FWHM) is broader and resolution of the peaks can only be ensured by the proper optimisation of the modulation amplitude.

**Figure 1:**
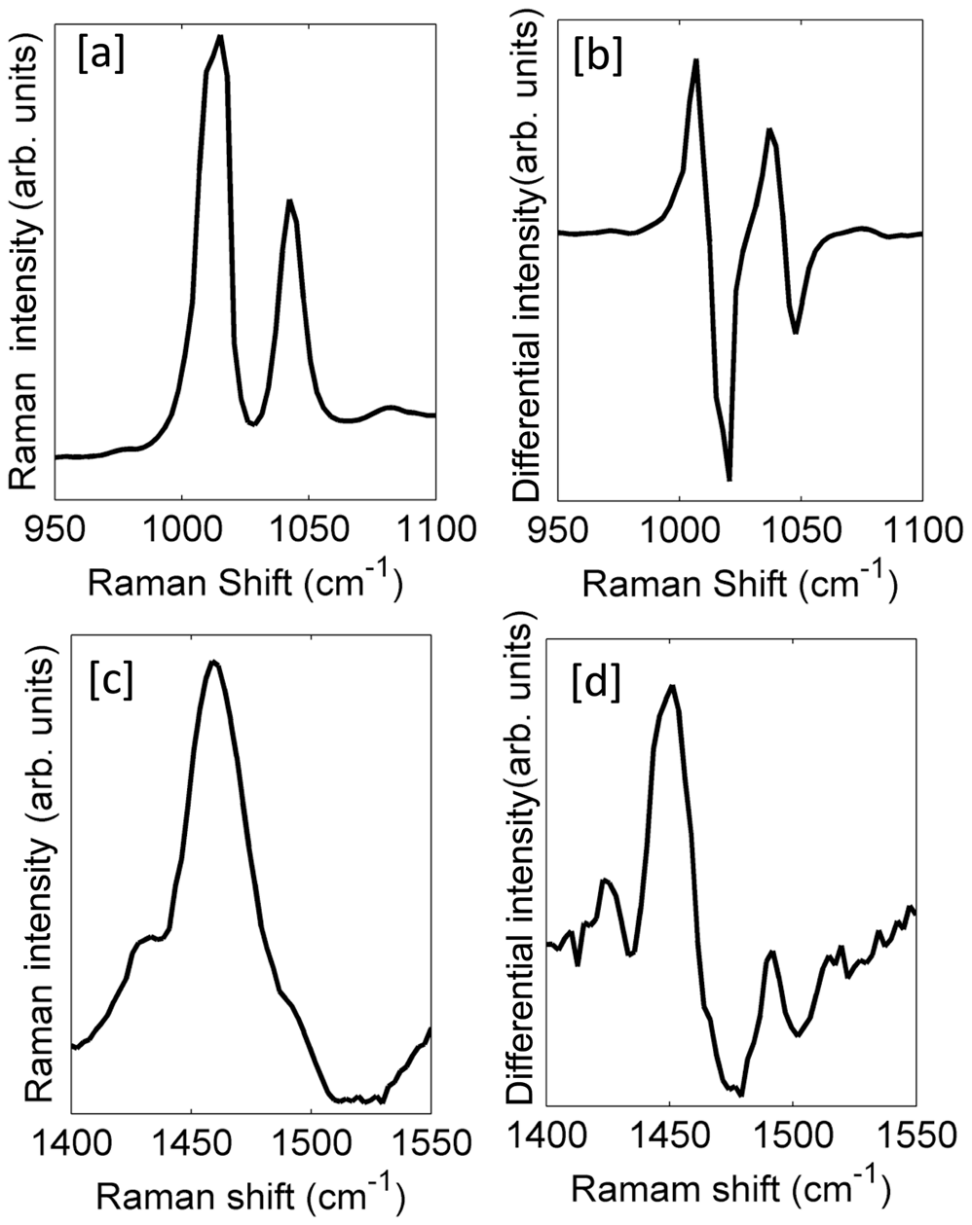
Illustration of the prominent peaks of polystyrene beads and bladder cancer cells (MGH-U1). [a] Standard Raman spectrum of the 1001.4 cm^−1^ peak of polystyrene [b] Wavelength Modulated (WM) Raman spectrum of the 1001.4 cm^−1^ peak of polystyrene [c] Standard Raman spectrum of the 1453 cm^−1^ peak of MGH-U1 [d] WM Raman spectrum of the 1453 cm^−1^ peak of MGH-U1.

Here we chose two different samples which differ significantly in their Raman cross-section strength and the FWHM. We chose polystyrene beads (∼ 20 µm) and Bladder Cancer cells (MGH-U1, ∼ 20 µm) for this experiment. The prominent peak at 1001.4 cm^−1^ for the polystyrene corresponding to the *ring breathing mode* and the peak at 1450 cm^−1^ for MGH-U1 corresponding to the *protein marker mod*e, shown in Figure 1, were chosen for this study [1], [25].

We adopted an empirical procedure to obtain the optimal modulation amplitude. The modulation amplitude was varied in discrete steps starting from Δν = 40 GHz which corresponds to Δλ = 0.08 nm to Δν = 240 GHz which corresponds to Δλ = 0.492 nm, in a symmetric trapezoidal pattern. The sampling rate was maintained at three for this study, for 12 cycles with a time constant of 5 s. The Signal to Noise Ratio (SNR) was estimated as the ratio of the peak to peak value of these two peaks to the standard deviation of the signal in a Raman band free spectral region ranging from 1750 cm^−1^ to 1800 cm^−1^. The SNRs were then plotted for varying modulation amplitudes.

**Figure 2:**
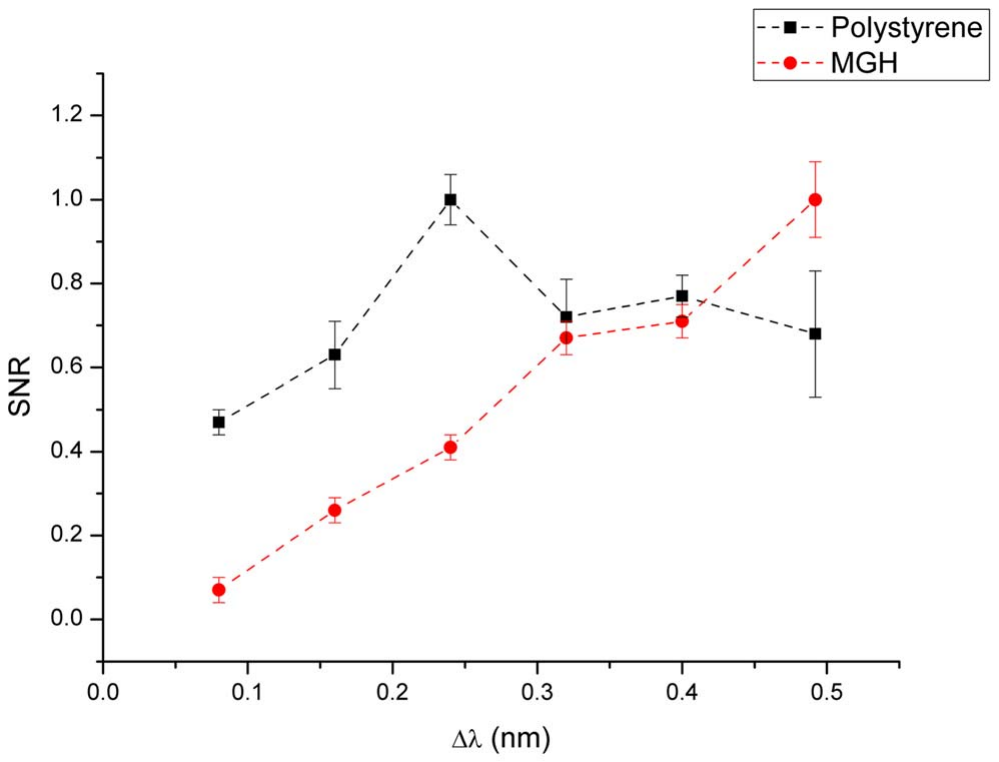
Characteristics of SNR variation with modulation amplitude for polystyrene and MGH-U1.

As can be seen from Figure 2, the SNR profile varies significantly between samples with high and low Raman cross-sections. The SNR peaks for the polystyrene sample at Δλ = 0.24 nm which corresponds to Δν = 120 GHz. This coincides with the modulation amplitude chosen for WMRS studies previously on relatively high Raman cross-section samples [14], [15]. In contrast to the case of polystyrene, the SNR recorded for MGH-U1 cells keeps on increasing as the modulation amplitude increases. The modulation range through which the experiment was performed was limited by the scanning range of the laser. For MGH-U1 cells, Δλ = 0.32 nm corresponding to Δν = 160 GHz was chosen for this study: this gives the minimum modulation amplitude that resolved the Raman bands whilst remaining well within the mode-hop free region of the laser. This study demonstrates that the modulation amplitude for WMRS would differ significantly between samples with differing Raman cross-section.

The next parameters to optimise were the sampling rate time constant and the number of cycles. For this study, the modulation amplitude was kept constant at Δλ = 0.32 nm (Δν = 160 GHz). The peak at 1450 cm^−1^ in the Raman spectrum of MGH-U1 cells was used to compare the variation in SNR for different parameters. Keeping the total acquisition time constant, Raman spectra of MGH-U1 cells were recorded for different sampling rate and time constant. The variation in SNR at a total acquisition time of 80 s for different sampling rate and time constant is shown in table 1. It can be seen that the SNR increases with increasing time constant.

**Table 1:** Illustration of the dependence of SNR on the time constant and the sampling rate.

Time constant →	0.5 s	1 s	5 s
Sampling rate ↓	(SNR)	(SNR)	(SNR)
**3**	62	70	90
**5**	55	82	91
**7**	51	69	88

The rows correspond to the sampling rate per cycle and columns correspond to the time constant.

**Figure 3:**
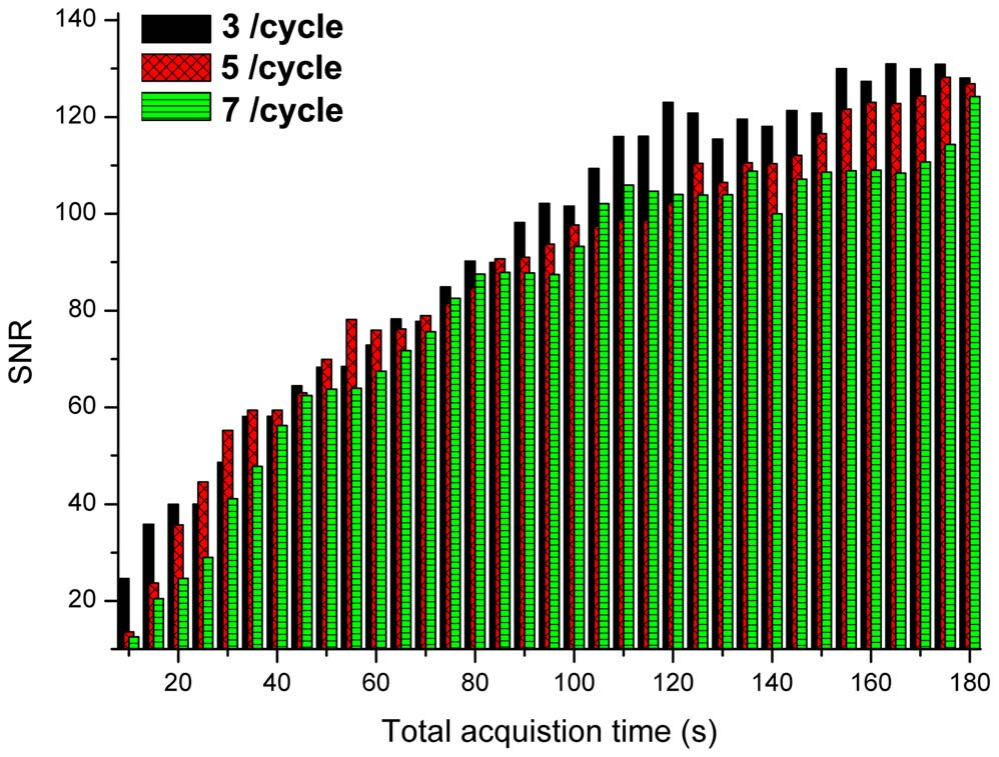
Illustration of the typical SNR characteristics. SNR characteristics, while varying the sampling rate, with progressing total acquisition time with a time constant of 5 s.

It is also seen in **Table 1** that the SNR does not significantly vary with differing sampling rate. To illustrate the effect of sampling rate on SNR, Figure 3 gives the variation of SNR while varying the total acquisition time for three cases of sampling rates at a time constant of 5 s. It can be seen that there is an increase in SNR with total acquisition time. Crucially, however the sampling rate does not make a significant contribution to the variation in SNR. This means the sampling rate can be kept minimal whilst an increase in the number of cycles increases the SNR of the observed Raman bands.

Based on these observations, experiments were performed to obtain optimized laser scanning parameters for cell discrimination at the highest throughput. This was performed by classification studies using PCA of WMRS spectra of normal urothelial cells (SV-HUC-1) and bladder cancer cells (MGH-U1).

**Figure 4:**
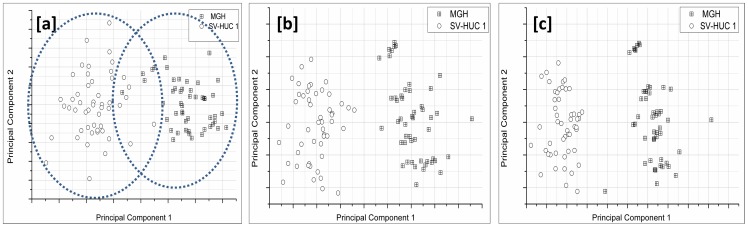
Cluster plot for MGH-U1 and SV-HUC 1. Plot of principal component 1 and principal component 2 of WM Raman spectra of 50 MGH-U1 cells and 50 control SV-HUC 1 cells for a time constant of 5 s, [a] clusters overlapping at a total acquisition time of 10 s, [b] segregated clusters at a total acquisition time of 15 s, [c] segregated clusters at a total acquisition time of 60 s.

For this study, the total acquisition time was varied, for constant modulation amplitude, keeping the time constant at 5 s. Figure 4 shows the PCA cluster plot of the Raman spectra acquired from MGH-U1 and SV-HUC-1 cell lines. As can be seen from Figure 4[a], at 10 s total acquisition time corresponding to 2 steps, the clusters overlap. With a 3 step sampling rate (15 s total acquisition time), the clusters were separated and the *separability* (defined here as the ratio of interclass variance to the intraclass variance) was 45% as shown in Figure 4[b]. This confirmed our earlier observation that WMRS yields a better sensitivity when compared to Shifted Excitation Raman Differential Spectroscopy (SERDS) [16]. Also Figure 2 shows that increase in sampling rate beyond 3 does not enhance SNR of the signal. This shows that a minimum of three different wavelengths for sampling is sufficient to discriminate between MGH-U1 and SV-HUC1.

Further we have explored the optimum number of cycles required for efficient discrimination. It was observed that increasing number of cycles, keeping sampling rate at 3 for a time constant 5 s did not alter the separability between the PCA clusters for SV-HUC-1 cells and MGH-U1 cells. An example is given in Figure 4[c], where it can be seen that the separability did not enhance significantly even for a total acquisition time that corresponds to 10 cycles. This shows that increasing number of cycles does not enhance the discrimination efficiency in this case, even though the SNR of the Raman bands may be higher with higher total acquisition time. Hence we found that the threshold SNR required to obtain efficient discrimination may be obtained with one acquisition cycle at a sampling rate of 3. Naturally the improvement in acquisition time will be cell dependent, yet our studies show the potential of our approach particularly for cell types where several minutes of signal acquisition, is the norm in Raman apparatus.

**Figure 5:**
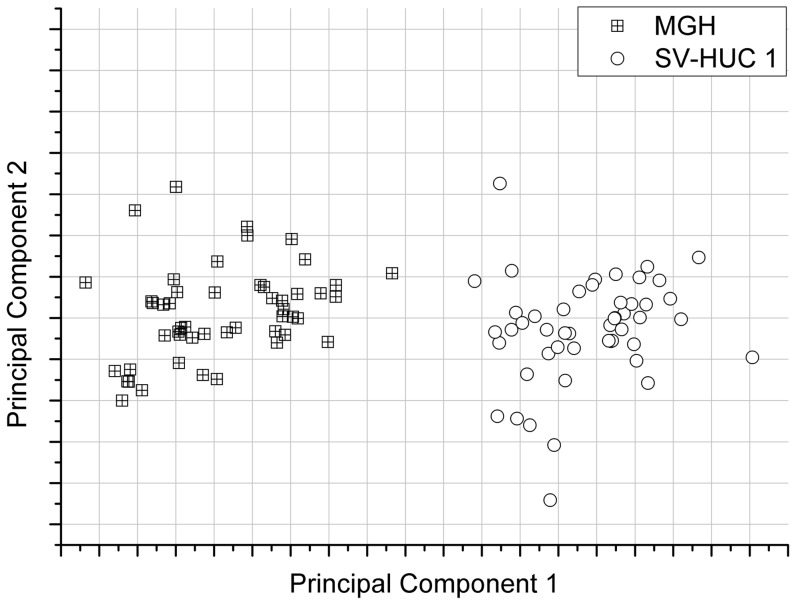
PCA of WM Raman spectra of 50 MGH-U1 cells and control SV-HUC 1. The time constant is 2 s and total acquisition time is 6 s.

Further we tried to find the minimum possible time constant that would give complete segregation of the cell samples as shown in Figure 5. We have observed that in this classification experiment, the minimum time constant, where the data was completely clustered was 2 s (corresponding to a total acquisition time of 6 s). For 1 s time constant, the clusters were not separated for any higher total acquisition time. Thus the optimised modulation parameters allowed a significant reduction in the total acquisition time to 6 s without compromising the discrimination efficiency. This is promising for high throughput cell screening using techniques such as flow cytometry for clinical applications.

### Conclusion

WMRS is a simple and reliable method for obtaining accurate Raman spectra. WMRS has been previously demonstrated to yield improved classification efficiency in comparison to standard Raman spectroscopy and thus holds promise for clinical applications. Further exploitation of this technique for useful high throughput applications requires a better understanding and optimisation of the modulation parameters. Here we have studied systematically the acquisition parameters of WMRS that affect the SNR of Raman bands. This allowed us to optimize the acquisition parameters to achieve minimum acquisition time. Our study demonstrated that, with a modulation amplitude exceeding Δλ = 0.32 nm (Δν = 160 GHz), the Raman bands of biological samples are resolvable. It was observed that the SNR of Raman bands increases with the time constant (single acquisition time) and the total acquisition time. The sampling rate did not affect the SNR of Raman bands as long as three or more wavelengths were sampled. This means that the optimum acquisition parameters would be the maximum modulation amplitude achievable within the mode-hop free region. The time constant required would depend on the Raman cross-section of the sample of interest. Using these optimized parameters, we have demonstrated complete discrimination of MGH-U1 and SV-HUC-1 cells with a total acquisition time of 6 s. This study defines a systematic protocol to optimise a WMRS system. This study will enable a wider uptake of WMRS for various biomedical applications and particularly open up new prospects for higher throughput Raman flow cytometry and sorting [26].
